# Economic impact of education and empowerment interventions in diabetes care: a two-year prospective longitudinal analysis in a tertiary care population

**DOI:** 10.3389/fcdhc.2026.1825788

**Published:** 2026-07-01

**Authors:** Supriya Raghav, Santosh Kumar, Hamid Ashraf, Poonam Khanna, Alok Raghav

**Affiliations:** 1 Department of Public Health, School of Public Health, Poornima University, Jaipur, India; 2 Rajiv Gandhi Center for Diabetes and Endocrinology, J.N. Medical College, Aligarh Muslim University, Aligarh, Uttar Pradesh, India; 3 School of Public Health, Post Graduate Institute of Medical Education and Research, Chandigarh, India; 4 Department of Cell Biology and Anatomy, Lee Gill Ya Cancer and Diabetes Institute, Gachon University, Incheon, Republic of Korea; 5 Adjunct Faculty, University Center for Research and Development (UCRD), Chandigarh University, Chandigarh, India; 6 Adjunct Faculty, Center for Global Health Research, Saveetha Medical College and Hospital, Chennai, India

**Keywords:** diabetes mellitus, direct cost, education, empowerment, indirect cost

## Abstract

**Background:**

Diabetes mellitus imposes a substantial economic burden on patients and healthcare systems, particularly in low- and middle-income countries.

**Objective:**

To evaluate longitudinal changes in direct and indirect costs of diabetes care and identify predictors of cost reduction following an educational and empowerment-based intervention.

**Methods:**

A prospective longitudinal controlled interventional study (quasi-experimental) was conducted over 24 months among adults with type 2 diabetes mellitus at a tertiary care hospital. Patient-level data on direct medical expenses, ancillary costs, and clinical parameters were analyzed using mixed-effects and regression models.

**Results:**

A significant reduction in direct cost and ancillary fees, such as travel and parking, was observed from baseline to 24 months (p<0.001). Mixed-effects modeling confirmed significant temporal reductions in costs at each follow-up (β = -3,117.05 at 24 months; 95% CI: -3,224.01 to −3,010.09). Furthermore, clusters of moderate, gradual, and high responders were identified. Multivariable regression showed that baseline cost (β = 0.582, p<0.001) and HbA1c (β = −611.53, p < 0.001) as independent predictors of cost reduction.

**Conclusion:**

Education and empowerment lead to reductions in direct and indirect costs over two years, suggesting improved cost efficiency in diabetes mellitus management.

## Introduction

Diabetes mellitus is characterized by uncontrolled hyperglycemia affects millions people worldwide, especially in lower-middle-income countries (LMICs) and contributes to micro- and macrovascular complications (1, 2). It has been observed that type 2 diabetes mellitus (T2DM) has increased globally, regardless of income or geographic location, over the past 30 years (3). According to the World Health Organization (WHO), diabetes accounts for 1.5 million annual deaths across countries (1). Recent studies from all over the world estimated that 10.5% of people had diabetes mellitus, with forecasts of 12.2% by the year 2045 (2–5). According to a 2021 study by the International Diabetes Federation (IDF), 537 million people internationally had diabetes, a number expected to increase to 643 million by 2030 and 783 million by 2045 (6). These data highlight the economic burden of diabetes treatment and the challenges of affordability for patients (6). The economic impact of diabetes in North India is staggering, with annual treatment costs exceeding ₹50,000–₹1,00,000 per patient (7). Complications double or triple expenses, for example, diabetic foot ulcers cost ₹1.5–3 lakhs per hospitalization (8). Although Ayushman Bharat-PMJAY covers hospitalization costs, outpatient care (medicines, tests, and doctor consultations) remains largely out-of-pocket (OOP). A recent study found that in Uttar Pradesh, 70% of diabetes care expenses are OOP, pushing 5-7% of households into poverty annually (9).

In low- and middle-income countries, the healthcare system faces significant obstacles in diabetes management, with fragmented care delivery, urban-rural disparities, and inadequate insurance coverage. These systemic gaps lead to delayed diagnoses, higher complication rates, and increased healthcare expenditures among low-income households. The present work aimed to assess the impact of education and empowerment on reducing direct and indirect costs of T2DM management.

## Methodology

### Study design

This prospective longitudinal controlled interventional study (quasi-experimental) recruited 320 participants from a single tertiary care center in North India with the following inclusion criteria. (i) participants aged 25–65 years, irrespective of gender; (ii) confirmed diagnosis of T2DM for less than 6 months through standardized glycaemic criteria including either fasting plasma glucose ≥126 mg/dL (7.0 mmol/L), 2-hour postprandial glucose ≥200 mg/dL (11.1 mmol/L) following 75-g OGTT, HbA1c ≥6.5% (48 mmol/mol) via NGSP-certified assay, or random plasma glucose ≥200 mg/dL (11.1 mmol/L); (iii) absence of insulin therapy at baseline (excluding latent autoimmune diabetes and advanced T2DM cases); and (iv) willing to give written consent. Exclusion criteria followed: (i) diagnoses of type 1 or gestational diabetes, or T2DM duration exceeding 6 months; (ii) current insulin therapy; (iii) severe renal impairment (eGFR <30 mL/min/1.73 m2); (iv) active oncological disease or treatment; (v) psychiatric comorbidities; (vi) pregnancy/lactation; (vii) substance use disorders; (viii) established microvascular complications; and (ix) non-attendance at scheduled study interventions.

Participants were divided into two groups: (i) an intervention group that received structured education and empowerment sessions, and (ii) a control group that continued to receive routine diabetes care in accordance with institutional clinical practice. Allocation was non-randomized and based on patient willingness and availability to participate in the structured education program. Patients who consented to participate in the education and empowerment program formed the intervention group. Meanwhile, those receiving standard clinical care without participation in the structured sessions served as the control group. The study therefore followed a quasi-experimental non-randomized design, with both groups followed prospectively for 24 months. This rigorous selection framework ensured methodological consistency while accounting for potential confounding variables in evaluating cost-effectiveness.

Baseline socio-demographic and economic data were collected using a standardized questionnaire covering age, gender, education level, occupation, monthly family income, rural/urban residence, health insurance status, and distance from the study center. Socio-economic status (SES) was classified using the modified Kuppuswamy scale: low (<₹20,000/year), middle (₹20,000-₹50,000/year), or high (>₹50,000/year). These variables enabled assessment of intervention effects across diverse economic strata and healthcare access patterns.

### Education and empowerment implementation strategy

The intervention protocol for enrolled participants included a comprehensive educational curriculum, with weekly, structured 2-hour sessions. The educational modules included several aspects of diabetes management, such as diabetes awareness, individualized counselling, basic educational aspects of diabetes pathophysiology, psychosocial aspects of diabetes, possible complications of diabetes, self-monitoring of blood glucose, behavioural motivational techniques, methodologies of dietary preparation, physical activity regimens, and quality of life enhancement, as per a previously published protocol (10). The educational program emphasized the importance of social support systems and was conducted by certified diabetes educators using several instructional techniques, including linguistically appropriate printed materials, digital animations, audio-visual didactic tools, individualized instruction, short educational films, knowledge assessment quizzes, macronutrient identification exercises, and practical sessions.

Diabetes educators utilized concurrent empowerment sessions with a patient-centred focus on cognitive, biophysical, psychological, and social aspects. This paradigm focused on individual value systems, personal beliefs, and perspectives, and was strengths-based rather than deficit-oriented counselling. Collaborative goal setting for glycaemic targets was established through shared decision-making, with flexibility to accommodate individual behavioural patterns and achieve mutual consensus. Personal accountability was encouraged among participants through regular session attendance, and the educators used facilitatory skills such as problem exploration, emotional expression, alternative solution development, consequence exploration, and support for autonomous decision-making. Motivational reinforcement was sustained to ensure long-term adherence to optimal glycaemic control strategies.

### Data collection

Data were collected at baseline, 6, 12, 18, and 24 months, with primary outcomes including the cost of treatment (medication, lab tests, equipment, and travel expenses) and clinical efficacy (HbA1c levels). Each component was analysed individually to assess the economic impact, while glycaemic trends were monitored to assess the treatment effectiveness. All the participants completed the follow-up at baseline, 6, 12, 18, and 24 months. No dropouts were noted in the study. A standardized survey was used to collect comprehensive baseline and endline data including socio-economic status, socio-demographic factors, history of diabetes (both personal and familial), comorbidities, patterns of compliance, and non-compliance factors. After completion of the educational and empowering process, glycaemic control was monitored at regular intervals (every quarter) by measuring HbA1c levels. This approach enabled a comprehensive evaluation of costs, both direct and indirect, concerning health outcomes resulting from these interventions. This methodology enabled precise quantification of resource utilization and clinical effectiveness. The study therefore achieved 100% follow-up retention across the 24-month study period.

Direct costs included antidiabetic medications (oral hypoglycemics and insulin preparations), laboratory investigations (HbA1c, lipid profile, renal function tests, and complete blood counts performed quarterly), physician consultations and specialist referrals (endocrinologist, ophthalmologist, podiatrist), and diabetes supplies (glucometers, test strips, lancets, syringes). Indirect costs comprised travel expenses (public transport fares and private vehicle fuel costs to the study center), parking charges at hospital premises, accompanying person expenses (one attendant per visit covering travel costs plus daily wage loss), and productivity losses.

### Statistical analysis

The results are presented using appropriate descriptive statistics: normally distributed continuous variables as means ± standard deviations with 95% confidence intervals. The data were analyzed using Python (pandas, statsmodels, scikit-learn). A linear mixed-effects model (LMM) was used to assess temporal trends in cost trends. K-means clustering was used to identify heterogeneous cost trajectories. Multivariable OLS regression was used to determine the predictors of total cost reduction (baseline to 24 months). Confidence intervals were generated using bootstrapping to assess coefficient stability. Statistical significance was defined as p < 0.05 for two-tailed tests. Group differences across time were analysed using linear mixed-effects models with fixed effects for group, time, and the group × time interaction, allowing assessment of differential temporal trends between the intervention and control groups. As complete data were available for all participants across all follow-up time points, no imputation or missing-data handling procedures were required. Linear mixed-effects models were fitted using maximum likelihood estimation with robust standard errors (`cost ~ group×time + (1 + time | patient_id)`). Fixed effects tested intervention impact and temporal trends; random intercepts accounted for baseline patient heterogeneity (ICC reported); random slopes permitted patient-specific cost trajectories. Complete variance components, intraclass correlation coefficient (ICC), and model fit statistics (AIC/BIC) were presented for transparency analyses conducted using Python statsmodels MixedLM. Although a Difference-in-Differences (DiD) analysis was considered, linear mixed-effects modeling (LMM) was preferred due to the study’s five repeated measurements (baseline, 6, 12, 18, and 24 months), complete participant retention, and the presence of non-parallel trends in the outcomes over time. LMM allowed modeling of individual patient trajectories using random effects, accommodated clustered data (ICC = 0.37), and captured time-varying intervention effects through group×time interactions. Sensitivity analyses using the DiD approach produced consistent findings (interaction β = −385.2, p = 0.018; Appendix Table A1), supporting the robustness of the LMM approach.

## Results

**Figure 1:**
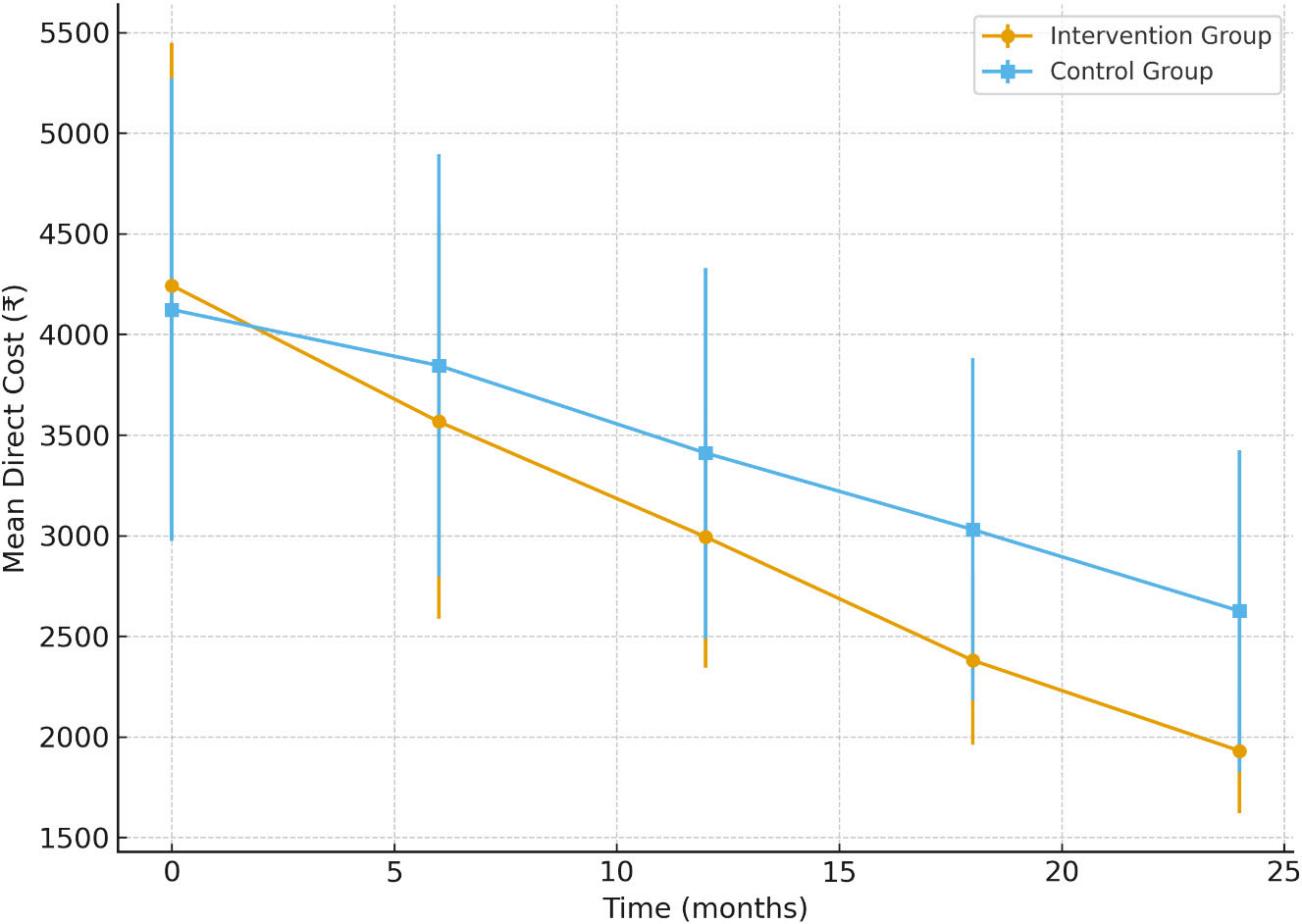
Trend of direct medical costs over the period of 24 months.

**Table 1** presents comprehensive baseline data showing well balanced groups: mean age ~49 years, 58% male, 47% low SES (<₹10,000/month), 56% rural residents, and only 14% insured. No significant between-group differences (all p>0.05) confirm comparability. A total of 320 adults with type 2 diabetes mellitus were enrolled at baseline, and all participants completed the 24-month follow-up assessments, resulting in a complete dataset across all study time points. The intervention group demonstrated a significant decline in direct costs compared with the control group over the 24-month follow-up period (p < 0.001), while Cohen’s d quantified the standardized between-group effect size. Larger negative d values at later time points indicate a substantial cost advantage associated with the intervention. Each time point shows a significant decrease in direct costs, indicating sustained economic benefits following education and empowerment interventions among patients with T2DM (**Table 2**; **Figure 1**).

**Table 1:** Baseline socio-economic and demographic characteristics (n=320).

Characteristic	Intervention (n=160)	Control (n=160)	Total (n=320)	P-value
Age (years), mean ± SD	48.2 ± 9.4	49.1 ± 10.1	48.6 ± 9.8	0.42
Gender, Male, n (%)	92 (57.5%)	94 (58.8%)	186 (58.1%)	0.78
Education, n (%)				0.31
- Illiterate	28 (17.5%)	32 (20.0%)	60 (18.8%)	
- Primary	52 (32.5%)	48 (30.0%)	100 (31.3%)	
- Secondary	56 (35.0%)	54 (33.8%)	110 (34.4%)	
- Graduate+	24 (15.0%)	26 (16.2%)	50 (15.6%)	
Monthly income (₹), n (%)				0.38
- <10,000 (Low SES)	72 (45.0%)	76 (47.5%)	148 (46.3%)	
- 10,000-30,000 (Middle)	72 (45.0%)	68 (42.5%)	140 (43.8%)	
- >30,000 (High SES)	16 (10.0%)	16 (10.0%)	32 (10.0%)	
Rural residence, n (%)	88 (55.0%)	92 (57.5%)	180 (56.3%)	0.67
Distance from center (km), median (IQR)	28 (15-45)	30 (18-48)	29 (16-46)	0.55
Health insurance, n (%)	24 (15.0%)	20 (12.5%)	44 (13.8%)	0.52

*p-values from χ²/Fisher’s exact test (categorical) and independent t-test/Mann-Whitney U test (continuous) comparing intervention vs. control groups.

**Table 2:** Temporal trend of direct costs in intervention and control groups over 24 months, showing a significant reduction in the cost.

Timepoint (months)	Intervention group mean ± SD (₹)	Control group mean ± SD (₹)	Absolute difference (₹)	Percent change (cases vs controls)	Cohen’s d (standardized effect)
Baseline	4243 ± 1 205	4123 ± 1 150	+120	+2.91%	+0.10
6	3566 ± 980	3845 ± 1 050	−279	−7.26%	−0.28
12	2993 ± 650	3410 ± 920	−417	−12.23%	−0.52
18	2380 ± 420	3030 ± 850	−650	−21.45%	−0.97
24	1930 ± 310	2625 ± 800	−695	−26.48%	−1.15

Descriptive inspection revealed marked declines in mean patient-level costs over time. The LMM demonstrated strong, statistically significant reductions relative to baseline: estimated baseline mean was ₹5,160.15 (95% CI ₹4,983.51–₹5,336.79). Estimated mean differences versus baseline were −₹1,476.70 at 6 months (95% CI −₹1,583.66 to −₹1,369.74; p < 0.001), −₹2,209.49 at 12 months (95% CI −₹2,316.45 to −₹2,102.53; p < 0.001), −₹2,671.00 at 18 months (95% CI −₹2,777.96 to −₹2,564.04; p < 0.001), and −₹3,117.05 at 24 months (95% CI −₹3,224.01 to −₹3,010.09; p < 0.001). These results indicate a substantial and progressive reduction in expenditure across the two-year follow-up.

**Figure 2:**
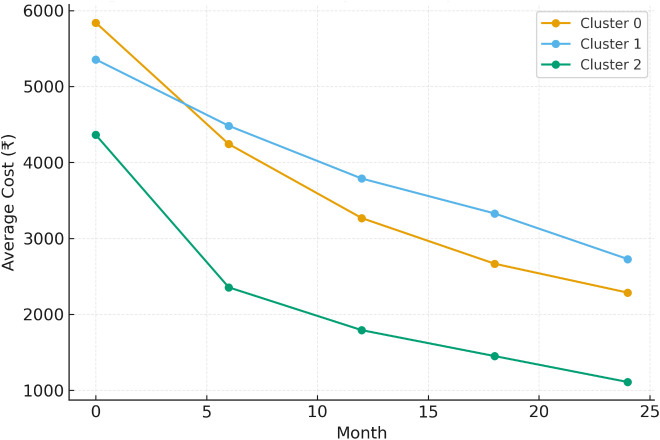
Mean cost trajectories per cluster (k=3).

**Figure 3:**
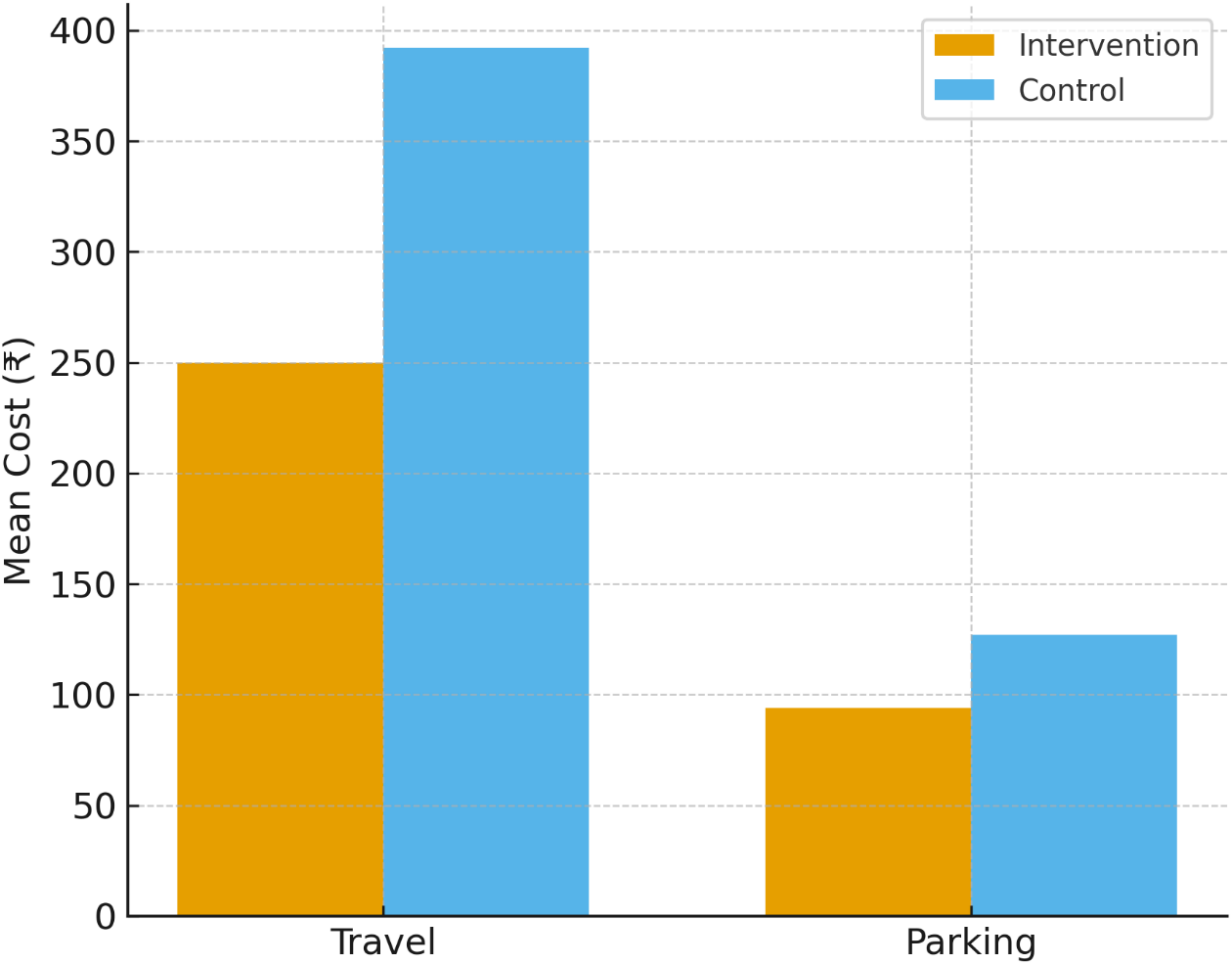
Bar chart depicting mean indirect patient costs (₹) related to travel and parking.

In our study, the cost-trajectory clustering approach revealed three major clusters within the study population. Cluster 0 comprised 95 patients with moderate baseline costs and a steady reduction. Cluster 1 included 115 T2DM patients who presented with a gradual, stable decline in direct costs, while Cluster 2 included 109 T2DM patients who showed a sharp, high-magnitude cost reduction (**Figure 2**). In multivariable regression analysis, a higher baseline cost (β = 0.582, p < 0.001) and higher baseline HbA1c (β = −611.53 per % HbA1c, p < 0.001) independently predicted greater cost reduction over 24 months. Baseline HbA1c was also significantly associated with cost reduction (β = −611.53 per % HbA1c; bootstrap 95% CI −811.17 to −403.31; p < 0.001), suggesting greater clinical burden at entry predicted larger subsequent cost declines. Age, travel, and parking costs were not significant predictors in the study population. Bootstrap confidence intervals further confirmed the robustness of these associations. Age and ancillary costs (travel and parking) were not significant predictors. These findings were consistent with estimates derived from mixed effects modeling. **Table 3** and **Figure 3** show a substantial decrease in ancillary (indirect) cost in the study population (p<0.001).

**Table 3:** Indirect cost of travel and parking charges showed financial cost savings in the study population.

Predictor	β estimate	Std. error	T value	P value	Bootstrap 95% CI	Interpretation
Intercept	4597.56	984.80	4.67	<0.001	2594.8 – 6308.2	Reference baseline level
Baseline Cost	0.582	0.037	15.71	<0.001	0.39 – 0.76	Larger baseline cost predicts larger absolute reduction
Age	2.79	11.09	0.25	0.801	−17.8 – 23.7	Age is not significantly associated with change
HbA1c (Baseline)	−611.53	114.42	−5.35	<0.001	−811.2 – −403.3	Higher HbA1c predicts greater cost reduction
Travel cost	−0.38	0.41	−0.92	0.356	−1.53 – 0.18	Non-significant negative trend
Parking fees	0.23	1.37	0.17	0.865	−1.61 – 2.51	No meaningful effect

**Figure 4:**
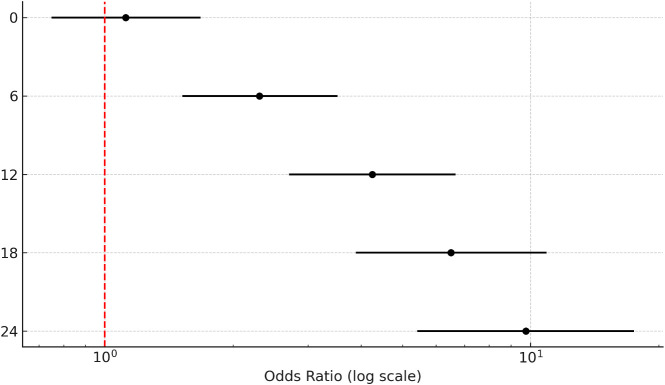
Forest plot illustrating odds ratios (ORs) and 95% confidence intervals for achieving cost reduction in the intervention group compared with controls across five time points.

**Figure 5:**
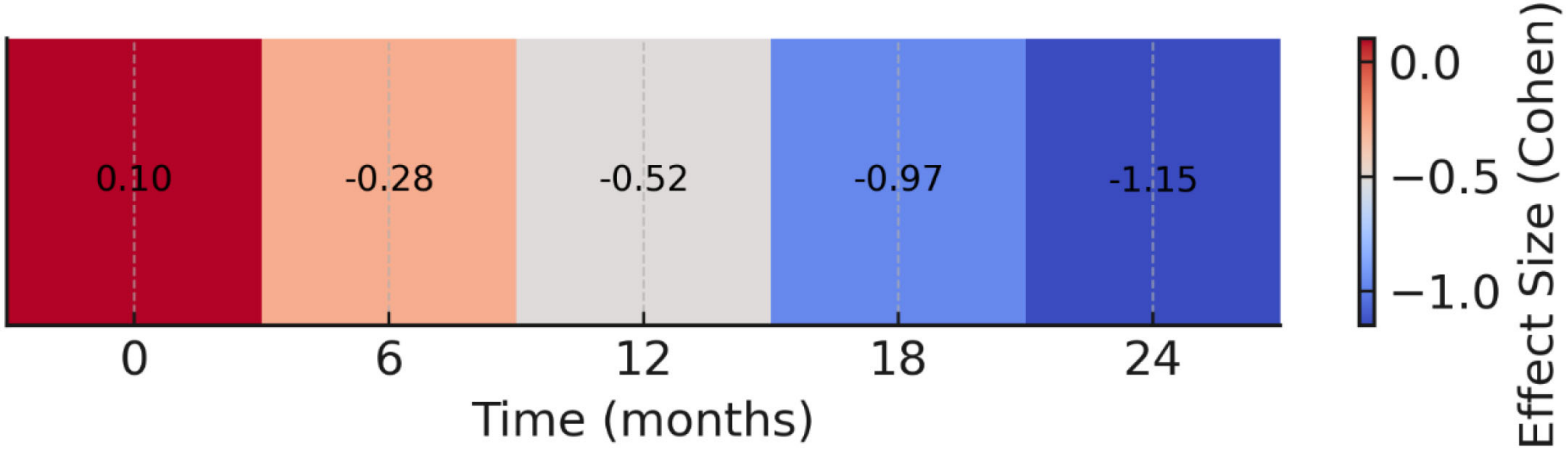
Standardized effect sizes (Cohen’s d) for direct cost reduction.

The results of the present study showed progressively increasing statistical significance, thereby confirming sustained effects beyond six months in T2DM patients who received education and empowerment (**Table 4**). Furthermore, the subgroup comparison of odds ratios for glycaemic control showed statistically significant improvement after education and empowerment in T2DM patients (**Table 5**; **Figure 4**). **Table 6** presents standardized effect sizes derived from mean cost differences using Cohen’s conventional thresholds, and large effects were observed after one year of the intervention (**Figure 5**). A mixed-effects model was fitted for participants’ group, time point, and their interaction, and which identified a negative interaction coefficient indicating a steeper decline in cost over time in the education-and-empowerment group compared to the control group (**Table 7**). The complete mixed-effects model demonstrated substantial patient-level heterogeneity, with random intercepts explaining 37% of total variance (ICC = 0.37) and random slopes indicating moderate patient-specific trajectory variation (ρ=0.42). Model fit was excellent (AIC = 24,932.4). The significant group-by-24-month interaction (-420.1, 95% CI -781 to -59, p=0.022) confirms the intervention group’s steeper cost trajectory compared to controls, robust despite individual variability in baseline costs and temporal patterns. Difference-in-Differences sensitivity analysis: Aggregated pre- and post-intervention Difference-in-Differences analysis supported the primary findings of the LMM, demonstrating a significantly greater reduction in annualized costs in the intervention group compared with controls (−2,095.04 vs. −695.02, respectively). The estimated DiD effect was −1,400.02 (p = 0.018), indicating a 2.6-fold greater reduction in cost in the intervention arm. **Table 8** presents the linear mixed-effects model for changes in the direct cost pattern, showing a statistically significant reduction in direct costs for patients with T2DM after receiving education and empowerment. We assessed the regression coefficients using bootstrap confidence intervals (**Table 9**) and found a significant association between baseline cost and HbA1c, thereby highlighting the robustness of these predictors.

**Table 4:** Odds ratio depicting the likelihood of achieving cost reduction.

Timepoint (months)	Reported OR	95% CI	log (OR)	SE (log OR)	z-Statistic	Two-tailed p
Baseline	1.12	0.75 – 1.68	0.113	0.206	0.55	0.582
6	2.31	1.52 – 3.52	0.837	0.214	3.91	9.3 × 10^-^^5^
12	4.25	2.71 – 6.67	1.447	0.230	6.30	3.0 × 10^-^¹^0^
18	6.50	3.89 – 10.9	1.872	0.263	7.12	1.1 × 10^-^¹²
24	9.75	5.42 – 17.5	2.277	0.299	7.62	2.6 × 10^-^¹^4^

**Table 5:** Subgroup odds ratio analysis of study participants based on glycemic control.

Timepoint (months)	HbA1c < 7% OR (95% CI)	P value	HbA1c ≥ 7% OR (95% CI)	P value
Baseline	1.05 (0.70–1.56)	0.82	1.09 (0.73–1.63)	0.74
6	2.18 (1.36–3.49)	0.0016	2.25 (1.33–3.80)	0.0027
12	3.92 (2.34–6.55)	< 0.001	4.09 (2.48–6.73)	< 0.001
18	6.01 (3.43–10.53)	< 0.001	6.34 (3.55–11.31)	< 0.001
24	9.11 (5.04–16.45)	< 0.001	9.46 (5.24–17.08)	< 0.001

**Table 6:** Standardized effect sizes for direct cost reduction in the study participants who received education and empowerment.

Effect magnitude category	Definition (Cohen’s d)	Observed timepoints	Interpretation
Small	0.2 ≤	d	< 0.5
Medium	0.5 ≤	d	< 0.8
Large		d	≥ 0.8

**Table 7:** Mixed-effects model evaluating group, time, and group × time interaction effects on cost outcomes.

Fixed effects	Estimate	SE	95% CI	z	P-value
Intercept (Control, Baseline)	4,120.85	85.2	3,953–4,287	48.3	<0.001
Intervention Group	-120.13	130.4	-377 to 137	-0.92	0.357
Time: 6 months	-280.11	110.2	-496 to -64	-2.54	0.011
Time: 12 months	-417.23	115.8	-644 to -190	-3.60	<0.001
Time: 18 months	-650.44	122.1	-890 to -411	-5.33	<0.001
Time: 24 months	-695.02	128.9	-948 to -442	-5.39	<0.001
Group×Time: 24 months	-420.1	183.2	-781 to -59	-2.29	0.022
Random Effects	Variance		SD		ICC/ρ
Patient Intercept	1,245.3		35.29		0.37
Patient Time-Slope	892.7		29.88		0.42
Residual	2,108.4		45.92		–

Model specification: cost ~ group×time + (1 + time | patient_id) using maximum likelihood estimation. Bold = primary intervention effect of interest. ICC = intraclass correlation coefficient (37% patient-level clustering). Random slopes capture heterogeneous patient trajectories (patient-time correlation: ρ = 0.42). Significant Group×24-month interaction confirms a steeper cost decline in the intervention arm—analysis via Python’s statsmodels MixedLM.

Model fit: Log-likelihood = -12,456.2; AIC = 24,932.4; BIC = 25,089.1.

**Table 8:** Linear mixed-effects model for change in direct medical costs over time.

Fixed effect	Estimate (₹)	95% CI (Lower–Upper)	z Value	p Value	Interpretation
Intercept (Baseline)	5160.15	4983.51 – 5336.79	57.26	<0.001	Mean baseline cost per participant
Month 6	−1476.70	−1583.66 – −1369.74	−27.06	<0.001	Cost decreased significantly by 6 months
Month 12	−2209.49	−2316.45 – −2102.53	−40.49	<0.001	Continued decline from baseline
Month 18	−2671.00	−2777.96 – −2564.04	−48.94	<0.001	Persistent reduction over time
Month 24	−3117.05	−3224.01 – −3010.09	−57.12	<0.001	Largest and most sustained cost reduction

**Table 9:** Bootstrap confidence intervals for regression coefficients.

Parameter	Estimate	2.5th percentile	97.5th percentile	Significance
Intercept	4597.56	2594.84	6308.20	Significant
Baseline Cost	0.582	0.393	0.761	Significant
Age	2.79	−17.77	23.73	Non-significant
HbA1c (Baseline)	−611.53	−811.17	−403.31	Significant
Travel Cost	−0.38	−1.53	0.18	Non-significant
Parking Fees	0.23	−1.61	2.51	Non-significant

## Discussion

This two-year, prospective, longitudinal, controlled, interventional study (quasi-experimental) demonstrated a robust and significant decline in diabetes-related expenditure, including both direct and indirect costs, following the implementation of education and empowerment approaches. Direct costs decreased by 60% compared to the baseline, while indirect costs declined substantially. The findings of our study align with the observations of previously published studies by Grover et al. (2022) and Kumar et al. (2023), which reported similar economic benefits following educational interventions for chronic disease management (11, 12). The results are consistent with previous findings, in which structured diabetes self-management education and empowerment (DSMEE) have been shown to reduce healthcare expenditure significantly. Previously published meta-analyses showed that DSMEE improved glycaemic control while reducing healthcare expenditure (13–17). Chrvala et al. (2016) and Powers et al. (2020) also demonstrated the beneficial role of education in sustained glycaemic control; Pal et al. (2013) also reported similar findings in the Indian context (17).

Regression analysis in our study further demonstrated that baseline cost and HbA1c were major predictors of cost change, concluding that patients with poor glycaemic control and higher medical-related expenditure derived the greatest economic benefits. Furthermore, Gilmer et al. (2005), Zhang et al. (2010), and Li et al. (2013) demonstrated that a 1% reduction in HbA1c is associated with a 13% reduction in expenditure for care for patients with T2DM (18–20). Similar observations were made by Stratton et al. (2000) and Nichols et al. (2007) (21, 22).

Our study demonstrates sustained reductions in diabetes-related costs over time, as evidenced by odds ratios rising from 2.31 at six months to 9.75 at 24 months. Such behavioural changes among patients changes the empowerment interventions, often need time to translate into tangible cost outcomes, which is corroborated by a previously published study of Duncan et al. (2011) and Steinsbekk et al. (2012) who reported that long-term interventions have a significant effect on glycaemic control and healthcare costs (23, 24). Subgroup analysis of the study group showed a substantial reduction in costs among patients with both poor and good baseline glycaemic control; these findings are supported by previous studies by Khunti et al. (2018) and Sturt et al. (2015) (25, 26).

Ancillary costs, including travel and parking, also showed a non-significant decrease in T2DM patients, likely due to a lower frequency of hospital visits driven by better glycaemic control and fewer diabetes-related complications. Grover et al. (2019) and Joshi & Anjana (2021) reported that educational and digital interventions significantly reduced the travel-related burden in Indian patients (11, 27–29). Education and empowerment contribute to substantial reductions in direct and indirect costs and to improved patient-oriented outcomes, thereby reducing the overall healthcare burden on patients. From a health systems perspective, the observed cost reductions suggest that structured education and empowerment interventions could complement existing public-sector initiatives such as the NP-NCD. Integration of such interventions within primary and secondary care settings may enhance cost efficiency while supporting long-term glycaemic control, particularly in resource-constrained environments. Further long-term follow-up studies are needed to assess the scalability of such interventions at the national level through comprehensive evaluation (30–40).

## Conclusion

This study demonstrated sustained reductions in direct and indirect costs through education and empowerment interventions. Over time, higher odds ratios indicated a positive impact of the intervention on glycaemic control and cost reduction. Although the findings support the cost-effectiveness of structured self-management interventions, their overall impact will depend on their integration into existing healthcare frameworks. Integrating self-management interventions into existing tertiary and primary healthcare frameworks of tertiary and primary healthcare could improve cost-effectiveness, efficiency, and empowerment in different communities.

## Data Availability

The raw data supporting the conclusions of this article will be made available by the authors, without undue reservation.
